# Dangers of Misappreciated or Overlooked Microbian Epidermitis

**Published:** 1919

**Authors:** 


**Dangers of Misappreciated or Overlooked Microbian Epidermitis. Prophylaxis of Erysipelas, Furunculosis etc., Through the Destruction of Cutaneous Microbian Nests.** By Dr. H. GOUGEROT. *Paris Médical,* November 2, 1918.

The author insists upon the necessity of giving immediate and adequate treatment to microbian dermo-epidermitis, which, if properly attended to, can be cured within a short time; but which, if overlooked, may cause partial or total incapacity of the patient, prevent surgical intervention, develop into erysipelas, etc.

*Erysipelas and Chronic Dermo-Epidermitis.* In some cases, acute erysipelas causes chronic epidermitis; in others, very slight chronic streptococcic epidermitis, such as pityriasis faciei or seborrheic eczema, following erysipelas, determines periodical outbreaks of the disease. Finally, erysipelas also occurs during the development of chronic dermo-epidermitis; the chronic infection, in these cases, develops into acute erysipelas, and the latter, through sensitization, prepares the organism for chronic epidermitis.

*Dangers of Sensitization.* As was proved by experimental inoculations, sensitization is either local or general. It is, as a rule, stronger in the epidermis surrounding the wound than in the rest of the body. In cases of local sensitization, epidermitis develops chiefly in the affected area, while in strong general sensitization or in a series of local sensibilized areas, the whole body may become infected by auto-inoculation. The sensitizing influence of a deep abscess has in some cases caused a recrudescence in local outbreaks of epidermitis.

*Prophylaxis of Cutaneous Infections by the Destruction of Microbian Nests.* The slightest manifestations of streptococcic or other epidermitis should be immediately checked, so as to avoid sensitization of the organism and further acute infectious outbreaks. All cases of chronic dermatosis should be carefully investigated and adequate treatment given.

The treatment for microbian dermo-epidermitis has been described by the author in a previous report. See " Traitement des Dermo-Epidermites Microbiennes de Guerre " : Gougerot. *Paris Médical*, January 1st, 1917.

